# Bonding Properties of Basalt Fiber and Strength Reduction According to Fiber Orientation

**DOI:** 10.3390/ma8105335

**Published:** 2015-09-30

**Authors:** Jeong-Il Choi, Bang Yeon Lee

**Affiliations:** School of Architecture, Chonnam National University, 77 Yongbong-ro, Buk-gu, Gwangju 61186, Korea; himancji@naver.com

**Keywords:** chemical bond, frictional bond, orientation, tensile strength, basalt fiber

## Abstract

The basalt fiber is a promising reinforcing fiber because it has a relatively higher tensile strength and a density similar to that of a concrete matrix as well as no corrosion possibility. This study investigated experimentally the bonding properties of basalt fiber with cementitious material as well as the effect of fiber orientation on the tensile strength of basalt fiber for evaluating basalt fiber’s suitability as a reinforcing fiber. Single fiber pullout tests were performed and then the tensile strength of fiber was measured according to fiber orientation. The test results showed that basalt fiber has a strong chemical bond with the cementitious matrix, 1.88 times higher than that of polyvinyl alcohol fibers with it. However, other properties of basalt fiber such as slip-hardening coefficient and strength reduction coefficient were worse than PVA and polyethylene fibers in terms of fiber bridging capacity. Theoretical fiber-bridging curves showed that the basalt fiber reinforcing system has a higher cracking strength than the PVA fiber reinforcing system, but the reinforcing system showed softening behavior after cracking.

## 1. Introduction

Experimental trials and patents involving the use of discontinuous steel reinforcing elements such as nails, wire segments, and metal chips to improve the properties of concrete have been ongoing since 1910 [1]. During the early 1960s in the United States, the first major investigation was made to evaluate the potential of steel fibers as a reinforcement for concrete [2]. Since then, a substantial amount of research, development, experimentation, and industrial application of steel or synthetic fiber reinforced concrete has occurred [3].

In particular, engineered cementitious composites (ECC) have been developed to improve the toughness of quasi-brittle cement-based materials such as concrete and mortar. ECC is a micromechanically-designed cementitious composite that is able to exhibit extreme tensile strain capacity (typically more than 3%) while requiring only a moderate amount of fibers (typically less than 2% in volume fraction) [4,5,6,7]. Polyethylene (PE) or polyvinyl-alcohol (PVA) fibers with high aspect ratio over 300 and a high tensile strength over 1,000 MPa are generally used for ECC. 

According to the Association Française de Génie Civil [8], ultra-high performance concrete (UHPC) tends to have the following properties: a compressive strength that is greater than 150 MPa, internal fiber reinforcement that ensures non-brittle behavior, and a high binder content with special aggregates. Furthermore, UHPC tends to have a very low water content and can achieve sufficient rheological properties through a combination of optimized granular packing and the addition of high-range water reducing admixtures. Straight steel fiber of 0.2 mm in diameter is generally used for UHPC. The tensile strength of UHPC also ranges from 12.0 to 19.1 MPa while the tensile strain capacity ranges from 0.3% to 0.79 % in 28 days [9,10]. 

Table 1 lists the typical physical properties of steel and synthetic fibers in high-performance fiber-reinforced cementitious composites and UHPC, as well as in basalt fiber [6,10,11,12]. The steel fiber used in UHPC has a high aspect ratio of about 100 and a high strength up to 2500 MPa. However, there are drawbacks in that the steel fiber has a potentially high corrosion possibility and its density is three times higher than that of a concrete matrix, which results in the sinking of steel fiber during mixing and casting, and finally, a poor fiber dispersion. Although the synthetic fiber has no corrosion possibility, its density is about 0.5 times that of the concrete matrix and its cost is high. On the other hand, basalt fiber has a higher strength compared to other types of fibers and a similar density with the concrete matrix as well as no corrosion possibility. Furthermore, the basalt fiber can retain about 90% of the normal temperature strength up to 600 °C [13]. Previous studies reported that basalt fiber is effective for strengthening concrete and improving the fracture behavior of concrete [13,14,15]. However, previous studies mainly investigated the properties of the concrete composite. Although the bonding and pullout properties are important for the high ductile fiber reinforced composite, previous studies on the properties needed for a reinforcing fiber such as the bonding and pullout properties between the fiber and matrix are fairly limited. Therefore, this study experimentally investigated the bonding properties of basalt fiber, as well as the effect of fiber orientation on the strength of basalt fiber, in order to evaluate the basalt fiber’s suitability as a reinforcing fiber.

**Table 1:** Typical physical properties of fibers.

Type of Fiber	Tensile Strength (MPa)	Density (g/cm^3^)	Corrosion
Steel	2500	7.5	High possibility
Polyvinyl-Alcohol (PVA)	1620	1.3	Little possibility
Polyethylene (PE)	3000	0.97	Little possibility
Basalt	3000–4840	2.65	Little possibility

## 2. Theoretical Background of the Fibers’ Bonding and Orientation Effect on the Composite

Lin *et al.* derived a theoretical single fiber debonding and pullout model, *i.e.*, the relation between pullout load *P* and pullout length δ based on a stress analysis and energy balance principle shown in the following equation [16]:

(1) $$P\left(\text{δ}\right)=\sqrt{\frac{{\text{π}}^{2}E_{f}d_{f}^{3}\tau_{0}\left(1+\text{η}\right)}{2}\text{δ}+\frac{\pi^{2}E_{f}d_{f}^{3}G_{d}\left(1+\text{η}\right)}{2}}$$

where *E_f_* is the elastic modulus of the fibers, *d_f_* is the diameter of the fibers, τ_0_ is the frictional bond strength, *G_d_* is the chemical bond strength, and η is (*V_f_E_f_*)/(*V_m_E_m_*) where *V_f_* is the volume fraction of fiber, *V_m_* is the volume of matrix, and *E_m_* is the elastic modulus of the matrix. 

After full fiber debonding, the relation between *P* and δ is given by Equation (2), since only frictional bonding stress remains, without chemical bonding stress.

(2) $$P\left(\text{δ}\right)=\text{π}d_{f}\tau_{0}\left[1+\frac{\text{δ}-{\text{δ}}_{0}}{d_{f}}\text{β}\right]\left[l_{e}-\left(\text{δ}-{\text{δ}}_{0}\right)\right]$$

where δ_0_ is the pullout length for full debonding, β is the slip-hardening coefficient, and *l_e_* is the fiber-embedded length. In the slippage regime, the fiber load is resisted by frictional forces. The fiber can undergo sliding with either slip hardening, constant friction, or the slip-softening effect. The fiber is also characterized by the coefficient β, which is, respectively, positive, zero, or negative. Slip-hardening often occurs with polymer fibers. Because polymer fibers are not as hard as the surrounding matrix, they can be easily damaged and a jamming effect can take place inside the matrix. This can also lead to an increasing load resisting fiber pullout. This phenomenon can be very beneficial as long as the fiber tensile strength is not exceeded. Conversely, constant friction or slip-softening are often observed when the fiber hardness is higher than that of the surrounding matrix [17].

The effect of the fiber orientation, known as the snubbing effect, on the pullout load is expressed as Equation (3), which is an empirical relation [18,19]. This is because actual short fiber composite fibers are randomly oriented.

(3) $$P\left(\text{θ}\right)=P\left(0\right)e^{f\text{θ}}$$

where *f* is the snubbing effect coefficient and θ is the inclination angle of fiber.

The effect of fiber orientation on fiber *in-situ* strength is expressed as Equation (4) [17].

(4) $${\text{σ}}_{fu}\left(\text{θ}\right)=\sigma_{fu}\left(0\right)e^{-f^{\prime }\text{θ}}$$

where $f^{\prime }$ is the fiber strength reduction coefficient.

β, *f* and $f^{\prime }$ are empirical, curve-fitting parameters. They are determined by single fibers with straight or inclined orientation pullout tests. 

The effect of fiber orientation on the multiple fibers in the composite is taken into consideration in Equation (5) in the form of a probability density function for fiber orientation and single fiber pullout load *P*(θ, *L_e_*, δ).

(5) $${\text{σ}}_{B}\left(\text{δ}\right)=\frac{4V_{f}}{\text{π}d_{f}^{2}}\int_{0}^{\text{π}/2}\int_{0}^{L_{f}/2}P\left(\text{θ},\;L_{e},\text{δ}\right)p\left(\text{θ}\right)\cos\left(\text{θ}\right)dL_{e}d\text{θ}$$

where p(θ) is the probability density function for fiber orientation.

## 3. Materials and Methods

### 3.1. Materials 

Three types of fibers, *i.e.*, basalt, PVA and PE fibers were investigated. The physical properties of each fiber and the chemical composition of basalt fiber are listed in Table 2 and Table 3, respectively. The PVA fiber was coated with 1.2% oil. The tensile strength of the fiber was measured in the laboratory.

**Table 2:** Properties of fibers.

Type of Fiber	Diameter (μm)	Tensile Strength (MPa)	Density (g/cm^3^)	Elastic Modulus (GPa)	Length (mm)
Basalt	12	1,773	2.65	89	12
PVA	40	1,202	1.3	41	12
PE	12	2,757	0.97	110	12

**Table 3:** Chemical composition of basalt fiber.

SiO_2_	Al_2_O_3_	B_2_O	CaO	MgO	NaO + K_2_O	TiO_2_	Fe_2_O_3_ + FeO
48–59	15–18	< 1	6–9	3–5	4–5	0.8–2.3	7–12

### 3.2. Fiber Pullout Test 

The mixture proportion of the matrix for the fiber pullout test is listed in Table 4. The alkali-activated slag and fine silica sand with an average diameter of 100 μm were used as a binding material and aggregate, respectively. The compressive strength of the matrix was 42 MPa. 

**Figure 1:**
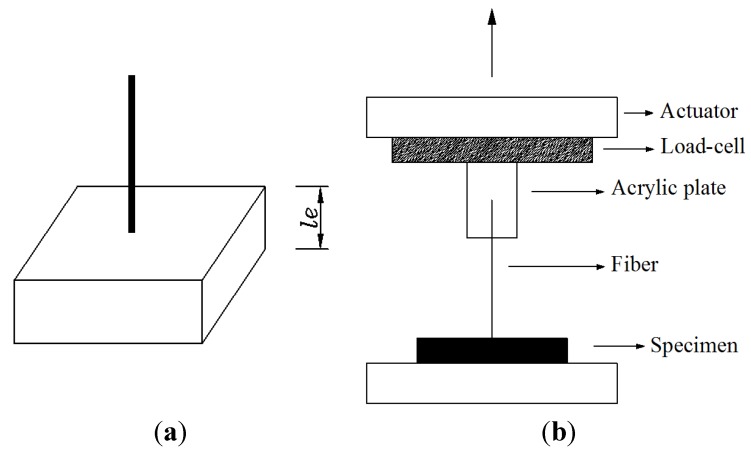
Specimen and test setup for fiber pullout test: (**a**) specimen and (**b**) test setup.

**Figure 2:**
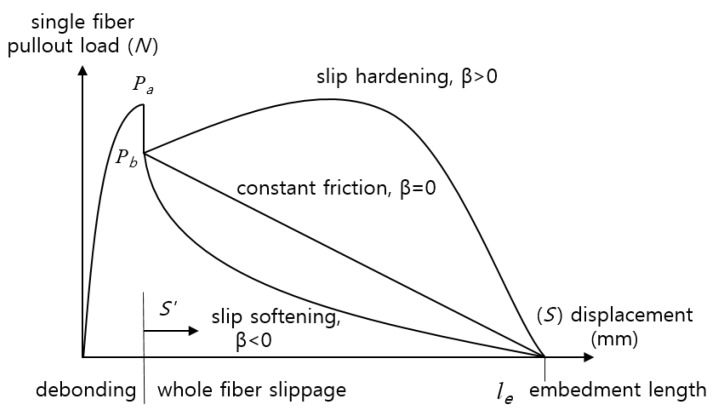
General profile of a single fiber pullout curve [17].

A single fiber pullout test was employed to measure the interfacial properties [17]. Figure 1 illustrates the specimen preparation and experimental setup. Four specimens were cast in a small acrylic mold. They were demolded in 2 days, followed by curing in water. The fiber embedment length was set at approximately 1.0 mm in an effort to ensure full debonding. The pullout tests were conducted on an electronic testing machine with the specimen configuration shown in Figure 1. A 5-N load cell was used to measure the pullout load of the fibers with a displacement rate of 0.1 mm/min. The chemical bond strength, frictional bond strength, and slip-hardening coefficient were calculated by using Equations (6)–(8), respectively.

(6) $$G_{d}=\;\frac{2{\left(P_{a}-\;P_{b}\right)}^{2}}{{\text{π}}^{2}E_{f}d_{f}^{3}}$$

(7) $$\tau_{0}=\;\frac{P_{b}}{\text{π}d_{f}l_{e}}$$

(8) $$\text{β}=\left(\frac{d_{f}}{l_{f}}\right)\left[\left(\frac{1}{\tau_{0}\text{π}d_{f}}\right)\left(\frac{∆P}{∆S^{\prime }}\right){|}_{s^{\prime }\to 0}+1\right]$$

where *P_a_* and *P_b_* are the pullout load from the single fiber pullout test (Figure 2).

**Table 4:** Mixture proportion of matrix (weight ratio).

Binder	Water	Silica Sand	Superplasticizer	Antifoamer
1	0.34	0.4	0.01	0.0002

### 3.3. Fiber Strength Test According to Fiber Orientation

**Figure 3:**
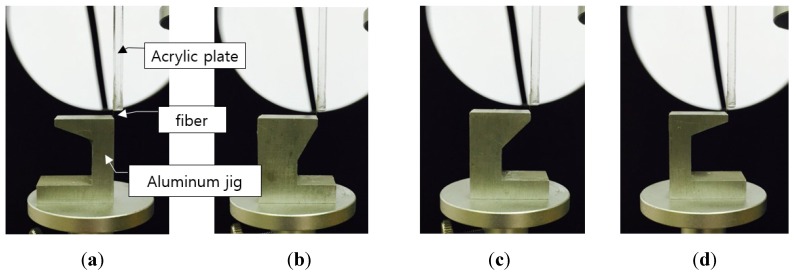
Test setup for measuring fiber tensile strength according to fiber orientation: (**a**) 0°; (**b**) 30°; (**c**) 45° and (**d**) 67.5°.

Fiber rupture behavior is dependent on the type of matrix. Therefore, fiber embedment in a matrix is necessary to reproduce fiber pull-and-rupture behavior in a certain composite. In this study, the test setup in Figure 3 was adopted to investigate the relative effect of fiber orientation on the fiber strength according to fiber types excluding the effect of the type of matrix. An aluminum jig was made and the one end of fiber was attached with glue at the jig and the other of fiber was attached to the acrylic plate. The fiber orientation was selected as 0°, 30°, 45° and 67.5°. A 5-N load cell was used to measure the fracture load of the fiber according to fiber orientation with a displacement rate of 0.1 mm/min. Based on the experimental results, the fiber strength reduction coefficient was determined by regression analysis. 

## 4. Results and Discussion

### 4.1. Bonding Property 

Table 5 lists the chemical bond, frictional bond, and slip-hardening coefficient of basalt and PVA fibers. The average chemical bond of the basalt fiber is 87.6% higher than that of PVA fiber. PVA fibers have polar hydroxyl (OH) groups and these groups attract water to them. Therefore, PVA fibers have hydrophilic interactions between the fiber and matrix. From the test results, it was determined that the basalt fiber has a stronger chemical bond strength between the fiber and matrix than the PVA fiber investigated in this study. This may be attributed to the similar chemical composition of the basalt fiber with the matrix, which results in a chemical reaction between the fiber and matrix. This micromechanical test supports the previous test results, *i.e.*, the improvement in the tensile strength of the composite in the previous study [14]. However, a high chemical bond can induce the brittle behavior of the composite by a concurrent fracture of the fiber with cracking. Similar phenomena were observed in Li’s study using PVA fibers without surface oil coating [5]. 

**Table 5:** Bonding properties.

Type of Fiber	$G_{d}$ (J/m^2^)	$\tau_{0}$ (MPa)	β
Basalt	2.59 ± 0.20	1.08 ± 0.19	0.0054 ± 0.0005
PVA	1.38 ± 0.29	1.05 ± 0.30	0.0221 ± 0.0032

The average frictional bond strength of the basalt fiber was 2.9% higher than that of the PVA fiber. On the other hand, the average slip-hardening coefficient of the basalt fiber was 24.4% compared with the PVA fiber. The slip-hardening coefficient means the degree of change of the frictional bond with slip. Thus, the fiber bridging capacity of the basalt fiber can decrease with slippage after cracking compared with that of the PVA fiber reinforcing system. Therefore, it is necessary to reduce the chemical bond strength by surface treatment and to increase the slip-hardening coefficient in order to increase the ductility of composites reinforced by basalt fiber.

### 4.2. Fiber Strength According to Fiber Orientation

**Figure 4:**
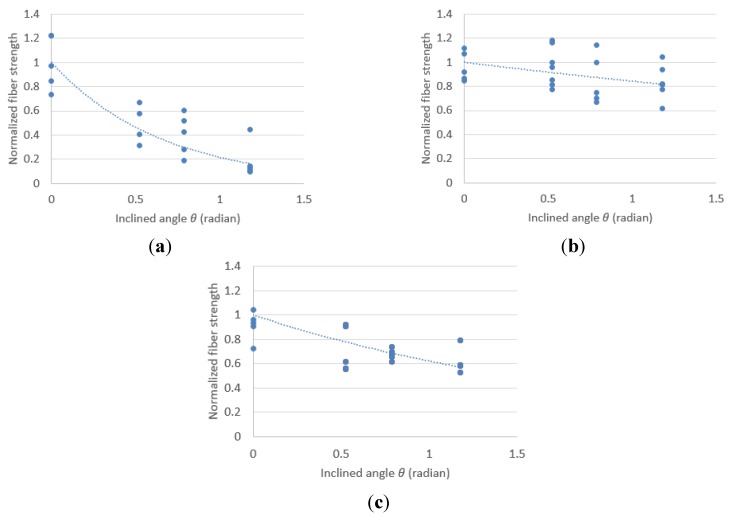
Normalized fiber strength with inclination angle: (**a**) Basalt fiber (**b**) polyvinyl-alcohol (PVA) fiber; and (**c**) polyethylene (PE) fiber.

Figure 4 shows the normalized fiber tensile strength with an inclination angle of fiber. The tensile strength of all fibers decreased with an increase in the inclination angle of fiber from 0° to 67.5° in all fibers. Table 6 lists the average fiber tensile strength of each fiber. Especially, it was observed that the strength reduction ratio of the basalt fiber is the highest among all the fibers investigated in this study. The average tensile strength of the PVA fiber was 1202 MPa at 0° and decreased by 7.3% at 30° compared to 0°. The average tensile strength of the PVA fiber also decreased by 15% and 17% at 45° and 67.5% compared to 0°, respectively. The PE fiber showed a higher tensile strength of 2757 MPa on average at 0° compared to the PVA fiber. The average tensile strength of the PE fiber decreased by 29%, 32% and 38% at 30°, 45° and 67.5°, respectively. Although the basalt fiber showed a higher tensile strength of 1778 MPa on average at 0° compared with the PVA fiber, the basalt fiber showed a higher reduction ratio compared with the PVA fiber and PE fiber. The average tensile strength of the basalt fiber decreased by 83% at 67.5°. 

**Table 6:** Average fiber tensile strength and strength reduction coefficient.

Fiber Type	0° (MPa)	30° (MPa)	45° (MPa)	67.5° (MPa)	$f^{\prime }$
Basalt	1773 ± 349	871 ± 247	715 ± 268	302 ± 219	1.535
PVA	1202 ± 132	1114 ± 182	1025 ± 223	1003 ± 161	0.171
PE	2757 ± 380	1962 ± 458	1867 ± 112	1697 ± 249	0.475

The strength reduction coefficients according to the inclination angle for each fiber based on Equation (4) by regression analysis are listed in Table 6. The average strength reduction coefficients of basalt fiber were nine times and three times higher than those of the PVA fiber and PE fiber, respectively. This may be attributed to the brittle behavior of the basalt fiber because the composition of the basalt fiber is basalt stone. Because a high strength reduction coefficient induces a large decrease in fiber strength with an inclination angle, the fiber bridging capacity of the basalt fiber can decrease compared with that of the PVA or PE fiber reinforcing system.

## 5. Analytical Study

**Figure 5:**
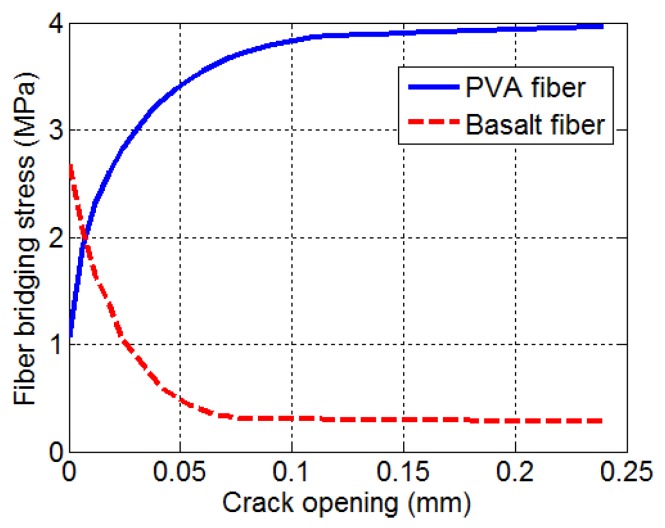
Predicted fiber-bridging curves.

Based on the interfacial properties determined by the fiber pullout and tensile tests, the theoretical fiber-bridging curves were calculated based on the fiber-bridging constitutive law σ(δ) and the numerical procedure for computing [20]. The micromechanical parameters used as model input for the basalt fiber and PVA fiber are listed in Table 7. The elastic modulus and spalling coefficient of the matrix were assumed to be 20 GPa and 500, respectively. Figure 5 shows the numerical analysis results. As was expected, the basalt fiber reinforcing system has a higher chemical bonding strength than the PVA fiber reinforcing system. This micromechanical analysis results support the experimental results, *i.e.*, strengthening the concrete and improving the fracture behavior of concrete [13,14,15]. However, the fiber bridging stress decreased with an increase of crack openings in the basalt fiber reinforcing system. This induces the strain softening behavior of the composite. On the other hand, fiber bridging stress increased with an increase of crack opening in the PVA fiber reinforcing system investigated in this study. From these analytical results, it is confirmed that the properties of basalt fiber should be tailored in order to increase the ductility of composites reinforced by basalt fiber. 

**Table 7:** Micromechanics parameters used as model input.

Type of Fiber	Tensile Strength (MPa)	Length (mm)	*d_f_* (μm)	*E_f_* (GPa)	*G_d_* (J/m^2^)	$\tau_{o}$ (MPa)	β	$f^{\prime }$	*f* *
Basalt	1773	12	12	89	2.59	1.08	0.0054	1.535	0.3
PVA	1202	12	40	41	1.38	1.05	0.0221	0.171	

* Assumed value [20].

## 6. Conclusions

This paper presents an experimental study on the bonding properties of basalt fiber and the effect of fiber orientation on the strength of basalt fiber. This was done to evaluate the suitability of basalt fiber as a reinforcing fiber. A single fiber pullout and tensile strength tests were performed in order to measure the bonding properties between a cementitious matrix and tensile strength according to fiber orientation. From the test results, it was determined that the chemical bond between the basalt fiber and alkali-activated mortar matrix with a compressive strength of 42 MPa is 87.6% higher than that of the hydrophilic PVA fiber with 1.2% oil coating. The frictional bond of the basalt fiber was 2.9% higher than that of the PVA fiber. On the other hand, the slip-hardening coefficient of the basalt fiber was 24.4% compared with PVA fibers. The tensile strength of the basalt fiber decreased with an increase in the inclination angle of fiber. The strength reduction coefficients of the basalt fiber were nine times and three times higher than those of the PVA fiber and PE fiber, respectively. Theoretical fiber-bridging curves showed that the basalt fiber reinforcing system has a higher cracking strength but it showed softening behavior after cracking. Therefore, this brittle behavior of basalt fiber can be a drawback in terms of the fiber bridging capacity when the fiber is not aligned, but randomly oriented, and it is necessary to reduce the chemical bond by surface treatment and to increase the slip-hardening coefficient in order to increase the ductility of composites reinforced by basalt fiber.
